# Significant differences in *FcγRIIa, FcγRIIIa and FcγRIIIb* genes polymorphism and anti-malarial IgG subclass pattern are associated with severe *Plasmodium falciparum* malaria in Saudi children

**DOI:** 10.1186/s12936-021-03901-0

**Published:** 2021-09-22

**Authors:** Amre Nasr, Ahmad Aljada, Osama Hamid, Hatim A. Elsheikh, Emad Masuadi, Ahmad Al-Bawab, Themer H. Alenazi, Amir Abushouk, Ayman M. Salah

**Affiliations:** 1 Department of Basic Medical Sciences, College of Medicine, King Saud Bin Abdulaziz University for Health Sciences (KSAU-HS), Riyadh, Kingdom of Saudi Arabia; 2 Department of Immunology, King Abdullah International Medical Research Centre (KAIMRC), Ministry of National Guard- Health Affairs, Riyadh, Kingdom of Saudi Arabia; 3 Department of Biochemistry and Molecular Medicine, College of Medicine, Alfaisal University, Riyadh, Saudi Arabia; 4 Department of Public Health, Jazan Health Affairs- District Ministry of Health, Jazan, Saudi Arabia; 5 Department of Pharmacology, College of Medicine, Taif University, POBox 888, Taif, 21944 Saudi Arabia; 6 Department of Medical Education, College of Medicine-Riyadh, King Saud Bin Abdul-Aziz University for Health Sciences, (KSAU-HS), Riyadh, Kingdom of Saudi Arabia; 7 Infectious Disease Division, Department of Medicine, King Abdulaziz Medical City, National Guard Health Affairs, RiyadhRiyadh, Saudi Arabia; 8 Department of Medicine, College of Medicine, King Saud Bin Abdulaziz University for Health Sciences (KSAU-HS), Riyadh, Kingdom of Saudi Arabia; 9 Department of Basic Medical Sciences, College of Medicine, King Saud Bin Abdul-Aziz University for Health Sciences, Jeddah, Kingdom of Saudi Arabia; 10 King Abdullah International Medical Research Centre (KAIMRC), National Guard Health Affairs, Jeddah, Kingdom of Saudi Arabia; 11 Department of Clinical Laboratory Sciences, College of Applied Medical Sciences, King Saud Bin Abdulaziz University for Health Sciences (KSAU-HS), Jeddah, Kingdom of Saudi Arabia

**Keywords:** *Plasmodium*, *Falciparum*, Malaria, IgG subclass, AMA-1, Saudi, Children

## Abstract

**Background:**

The FcγRs genotypes have been reported to play a key role in the defence against malaria parasites through both cellular and humoral immunity. This study aimed to investigate the possible correlation between *FcγR *(*IIa*,* IIIa*,* and IIIb*) genes polymorphism and the clinical outcome for anti‐malarial antibody response of *Plasmodium falciparum* infection among Saudi children.

**Methods:**

A total of 600 volunteers were enrolled in this study, including 200 malaria-free control (MFC) subjects, 218 patients with uncomplicated malaria (UM) and 182 patients with severe malaria (SM). The *FcγR* genotypes were analysed using PCR amplification methods, and measurements of immunoglobulin were determined using enzyme-linked immunosorbent assay (ELISA) technique.

**Results:**

The data revealed that the *FcγRIIa-R/R131* showed a statistically significant association with SM patients when compared to UM patients. Furthermore, higher levels of IgG1, IgG2, and IgG4 were associated with the *FcγRIIa-H/H131* genotype among UM patients. Although the *FcγRIIa-F/V176* genotype was not associated with UM, it showed a significant association with severe malaria. Interestingly, the *FcγRIIIa-V/V176* genotype offered protection against SM. Moreover, SM patients carrying the *FcγRIIIa-F/F* genotype showed higher levels of AMA-1-specific IgG2 and IgG4 antibodies. The *FcγRIIIb-NA1/NA1* and *FcγRIIIb-NA2/NA2* genotypes did not show significant differences between the UM and the MFC groups. However, the genotype *FcγRIIIb-NA2/NA2* was statistically significantly associated with SM patients.

**Conclusions:**

The data presented in this study suggest that the influence of the *FcγRIIa-R/R131, FcγRIIIa-F/F176* and *FcγRIIIb-NA2/NA2* genotypes are statistically significantly associated with SM patients. However, the *FcγRIIa-H/H13* and *FcγRIIIa-V/V176* genotypes have demonstrated a protective effect against SM when compared to UM patients. The impact of the *FcyR *(*IIa*,* IIIa and IIIb*) gene variants and anti-malaria IgG subclasses play an important role in susceptibility to malaria infection and disease outcome in Saudi children.

## Background

Malaria is a parasitic infectious disease caused by five species of *Plasmodium* that are transmitted to humans via mosquito bites. Of these species, *Plasmodium falciparum*, which is most prevalent in Africa, and *Plasmodium vivax* pose the greatest threat to health. In Saudi Arabia, *P. falciparum* represents about 99% of the total cases of malaria, while only 1% of patients are infected by *P. vivax*. In 2019, malaria affected about 229 million worldwide and contributed to 409,000 deaths. Children below the age of five were amongst the most vulnerable groups affected by the disease [1]. According to the WHO figures, between 2010–2015 in Saudi Arabia, the number of recorded malaria patients was steadily below 100, but it rose to 272 cases in 2016. This was mostly due to increased migration of people from war zones along the border with Yemen, as well as difficulties in providing adequate medical services in those regions. However, the health service in this country remains vigilant and offers free diagnosis and treatment for all patients.

Given the increase in the number of malaria infections and its apparent threat to people’s lives; there was a call for further studies that can assess individual’s susceptibility. This led to the current study in which the authors looked at genes (FcγR) within the innate immunity that are responsible for receptor expression on immune cells (including macrophages, neutrophils, NK cells). These cell receptors have the ability to recognize certain antibodies that will bind to antigens, such as antigens of *P. falciparum*.

There are three sub-families of surface receptors for the Fc region of the IgG, designated as FcγRI, II, and III [2]. Most immune cells express Fc receptors that are crucial for determining the specificity of IgG antibodies [3]. FcγR induces monocyte activation features such as phagocytosis, degranulation, superoxide generation, antibody-dependent cell inhibition, cytokine production, and antibody regulation, which are essential for host defence and immune regulation [4, 5]. The effectiveness of IgG‐induced FcγR activity demonstrates inter‐individual heterogeneity due to the genetic polymorphisms of the three subclasses of FcγR; *FcγRIIa* (CD32a), *FcγRIIIa* (CD16a), and *FcγRIIIb* (CD16b) [5].

Previous studies showed that several polymorphisms have been detected in the Fcγ genes encoding these receptors (FcγRs), associated with susceptibility or resistance to malaria outcome in different populations [3, 4, 6–12]. A recent review by Amiah et al. [13] described the FcγRs polymorphisms and the impact of these variations on the response of the host to infection. It also provided new perspectives for the potential design of an effective malaria vaccine [13].

The present study aimed to investigate the possible relationship between the expression of *FcγRIIa* (CD32a), *FcγRIIIa* (CD16a), and *FcγRIIIb* (CD16b) gene variants and the antibodies against the malarial apical membrane antigen 1 (AMA-1) in association with the susceptibility to malaria infection among Saudi children.

## Methods

### Study area

This study was conducted at Bani Malik General Hospital in Jazan Region (BMGHJ), located in the Southern part of the Kingdom of Saudi Arabia (KSA), during three transmission seasons from October 2015 to March 2018. The highlights of this study setting have already been described in related previous studies [3, 14–16].

### Study design and patients

A prospective case–control study was conducted in children attending the outpatient clinic of BMGHJ, with a confirmed clinically diagnosed *P. falciparum* infection. Patients with positive thick blood film for *P. falciparum* asexual parasites were recruited based on the microscopic diagnosis.

Participants with no features of severe malaria were defined as having uncomplicated *P. falciparum* infection. Children were diagnosed with severe malaria on the basis of one or more of the following: severe malarial anaemia, cerebral malaria, hypoglycaemia, jaundice, acidosis, acute kidney injury (renal impairment), significant bleeding, pulmonary oedema, and shock as described in detailed by the World Health Organization (WHO) [17]. These clinical manifestations occurred in the absence of any identifiable alternative cause other than *P. falciparum* asexual parasitaemia. Children with cerebral malaria had a Blantyre Coma Score (BCS) < 3 at 4 h post-admission. Children with severe malarial anaemia had a blood haemoglobin concentration of ˂ 5 g/dL or a haematocrit value of < 15% together with a parasite count above 10,000/μL. All other children recruited in the study had a haemoglobin concentration above this level. The control group were selected from the Child and Woman Health Clinics (CWHC) that provide children health services including routine vaccinations, as well as providing seasonal vaccines for children. Once the sample was collected, it was matched for age, gender, and ethnicity. Enrolment to the control group was confirmed following a physical clinical examination to ensure that the children did not have serious illnesses or any signs/symptoms of malaria according to information provided by parents/guardians.

The study excluded children with multiple severe malaria complications or any co-infectious diseases. None of the participants were positive for HIV. All the children were recruited during three malaria transmission seasons from October 2015 to March 2018.

### Sample collection

After the diagnosis of malaria and before the start of the pharmacological course of treatment; 100 µL of blood was spotted and dried on filter paper (Qualitative filter paper, Grade 1, circles, diam. 42.5 mm from Whatman®, Sigma-Aldrich®). This collected sample was used for investigating Fcγ receptor gene polymorphism, parasite detection using PCR, and measurement of immunoglobulins as described earlier [18, 19].

### Serum elution from filter-paper samples

To elute dried samples from filter-paper, a hole puncher of ϕ 6 mm was used for punching out filter-paper discs and placed in Eppendorf tubes with 100 µL of phosphate-buffered saline (PBS). Subsequently, the discs were transferred onto 10 mL tubes. Then, 500 µL of (PBS) with 0.05% Tween and 0.5% bovine serum albumin (BSA) were added to the tubes and incubated under shaking for 2 h at room temperature. After incubation, the samples were vigorously shaken with a vortex for 30 s, and the supernatants containing the eluted sera were aliquoted in cryotubes (1.5 ml) and stored at − 20 °C till analysis. Each extracted sample contained an approximately 1:100 diluted serum [18].

### DNA extraction

DNA was extracted from 50 µL dried drop of blood sample on the filter paper using the QIAamp DNA Mini Kit (Qiagen®, Hamburg, Germany). The extracted DNA was re-suspended in a 150 µL of Tris–borate-EDTA (TBE) buffer.

### Parasite genotype

Detection of *P. falciparum* was based on targeting the *AMA-1_3D7* gene using polymerase chain reaction (PCR) from 5 µL of the extracted DNA samples [20].

### Enzyme-linked immunosorbent assays (ELISA)

IgG subclass antibodies were measured against the recombinant AMA-1 anti-malarial antigen. The total levels of IgG and its subclasses were measured using enzyme-linked immunosorbent assays (ELISA) as previously described in detail [11, 21], and as recently reported [22].

### Genotyping of FcγRs polymorphisms

The *FcγRIIa*-*131R/H* (rs1801274, assay ID: C__9077561_20) and *FcγRIIIa-176F/V* (rs396991, assay ID: C__25815666_10) polymorphisms were genotyped using the high-throughput TaqMan® 5′ allelic discrimination assay-by-design method, as per the instructions of the manufacturer (Applied Biosystems, Foster City, CA, USA). The *FcγRIIIb*-*NA1/NA2* genotyping for the rs448740 (N65S) and rs147574249 (N82D) was carried out in accordance with the formerly described Restriction Fragment Length Polymorphism (RFLP) method [12, 23].

### Statistical analysis

Statistical analysis was done by SPSS statistical software version 23 for Windows (IBM© SPSS^©^ statistics). In this study, the median and 25% and 75% quartile of antibody (total IgG and IgG subclasses) levels were analysed using nonparametric (Kruskal–Wallis) tests and the *P* values were determined. With respect to the risk of malaria infection in children, all values of *P* < 0.05, 95% confidence interval (CI) for odds ratio (OR) that did not cross 1.00 were considered statistically significant. In the analysis, *FcγRIIa*-*R/H131* polymorphism was used as a reference, due to its utmost prevalence in humans [24]. Using the same software, a 2 × 2 chi-square test was used to compare the overall allele frequency. The *Hardy–Weinberg equilibrium *(*HWE*) for genotypic deviation was assessed using a chi-squared statistical test. The logistic regression analysis was performed to test for the association between the *FcγRs* genotypes related to higher levels of anti-malarial IgG subclass among severe malaria compared to uncomplicated malaria patients. Associations were quantified using OR with 95% CI that did not cross 1.00 with P value < 0.05, defined as statistically significant. As shown below; each IgG subclass was ranked in malaria-free control in two categories based on the levels of anti-malarial antibodies.

## Results

### Classification of the study participants

In this study, demographic data on malaria, parasite density, and disease complication variables were analysed for 600 children of matched gender and age. The 600 subjects were categorised into three different groups. Group I: The malaria-free control [MFC, n = 200 (33.3%) subjects]; included subjects without symptoms of the disease and showed negative results for blood film examination and PCR of the malaria parasites. Group II: Uncomplicated malaria [UM, n = 218 (36.3%) patients]. Group III: severe malaria [SM, n = 182 (30.3%) patients]. Group III included patients with severe malaria [n = 182 (30.3%)], (Table 1). The mean number of parasites in severe malaria patients was significantly higher compared to uncomplicated malaria, P < 0.001 (Table 1). The body temperature was significantly different between the study populations, P < 0.001 (Table 1).

**Table 1:** Description of study participants

Characteristics	Malaria-Free controls (MFC) n = 200	Uncomplicated malaria (UM) n = 218	Severe malaria (SM) n = 182	P value Between Groups
Gender				
Girls n = 321	104(52%)	115 (52.8%)	102 (56%)	0.704
Boys n = 279	96 (48%)	103 (47.2%)	80 (44%)	
Age (mean ± SD)	4.0 ± 1.4	4.1 ± 1.4	4.1 ± 1.1	0.89
Parasite density (mean ± SD)	NA	6438.4 ± 990	15,911.4 ± 403	< 0.001
Temperature (mean ± SD)	36.9 ± 0.28	38.9 ± 0.72	41.1 ± 0.7	< 0.001
Malaria complication				
Severe anaemia n (%)	NA	NA	56 (30.8%)	< 0.001
Cerebral malaria n (%)	NA	NA	61 (33.5%)	
Other complication^a^ n (%)	NA	NA	65 (35.7%)	

^a^Other complication (hypoglycaemia, jaundice, pulmonary oedema and acute respiratory distress)

### Comparison between the distribution of the *FcγRIIa* genotype and its allelic frequencies among the different study groups

The genotype frequencies for *FcγRIIa* did not deviate from the expectations of the HWE in each genotype group (Table 2). The frequencies for the individuals carrying the *FcγRIIa*-*R/R131* genotypes in UM group were lower than the ones in MFC. However, the logistic regression analysis revealed that there was no statistically significant difference between UM and MFC amongst both genotypes *R/R131* [18.0% in UM versus 15.1% in MFC; OR = 1.39, 95% CI (0.89 to 2.19) and *P* value = 0.15] (Table 3). In contrast, the genotypes *FcγRIIa*-*H/H131* were statistically significantly higher in UM compared to MFC groups [36.7% in UM versus 30% MFC; OR = 0.92, 95% CI (0.62 to 1.36) and *P* value = 0.026] using the heterozygotes as the reference group (Table 3). The *FcγRIIa*-*R/R131* genotype was statistically significant associated with SM compared to UM [34.6% in SM versus 15.1% in UM; OR = 2.132, 95% CI (1.287 to 3.533) and *P* value = 0.003]. In contrast, the *FcγRIIa*-*H/H131* genotype was negatively associated with SM compared to UM [13.7% in SM *versus* 36.7% in UM; OR = 0.349, 95% CI (0.206 to 0.592) and *P* value < 0.001] (Table 3). The frequencies of the *FcγRIIa*-*H/R131* genotype were almost the same among the three groups of MFC, UM, and SM (52.0%, 48.2%, and 51.6%, respectively) (Table 2).

**Table 2:** Distribution of FcγRIIa, FcγRIIIa and FcγRIIIb genotypes and alleles frequency in the different study groups

Genotypes	Malaria-free controls (n = 200)	Uncomplicated malaria (n = 218)	Severe malaria (n = 182)
FcγRIIa			
R/R (%)	36 (18.0%)	33 (15.1%)	63 (34.6%)
H/R (%)	104 (52.0%)	105 (48.2%)	94 (51.6%)
H/H (%)	60 (30.0%)	80 (36.7%)	25 (13.7%)
Allele frequency			
R allele	0.44	0.40	0.61
H allele	0.56	0.60	0.39
*HWE*^a^	0.44	0.87	0.28
FcγRIIIa			
F/F (%)	91 (45.5%)	85 (39.0%)	132 (72.5%)
F/V (%)	80 (40.0%)	105 (48.2%)	43 (23.6%)
V/V (%)	29 (14.5%)	28 (12.8%)	7 (3.8%)
Allele frequency			
F allele	0.65	0.63	0.84
V allele	0.35	0.37	0.16
*HWE*^a^	0.10	0.62	0.15
FcγRIIIb			
NA1/NA1 (%)	34 (17.0%)	33 (15.1%)	18 (9.9%)
NA1/NA2 (%)	92 (46.0%)	105 (48.2%)	70 (38.5%)
NA2/NA2 (%)	74 (37.0%)	80 (36.7%)	94 (51.6%)
Allele frequency			
NA1 allele	0.6	0.39	0.29
NA2 allele	0.4	0.61	0.71
*HWE*^a^	0.56	0.88	0.36

^a^If < 0.05—not consistent with *Hardy–Weinberg equilibrium “HWE”*

**Table 3:** Association between individual FcγRIIa, FcγRIIIa and FcγRIIIb genotypes and severity of malaria

	UM versus MFC		SM versus UM	
	Adjusted^a^ OR (95% CI)	P value	Adjusted^b^ OR (95% CI)	P value
FcγRIIa				
R/R	1.39 (0.89–2.19)	0.15	2.132 (1.287–3.533)	0.003
H/R	1.00		1.00	
H/H	0.92 (0.62–1.36)	0.026	0.349 (0.206–0.592)	< 0.001
FcγRIIIa				
F/F	1.95 (0.65–2.38)	0.79	11.51 (6.71–19.77)	< 0.001
F/V	1.00		1.00	
V/V	1.72 (1.04–2.82)	0.13	0.20 (0.09–0.47)	< 0.001
FcγRIIIb				
NA1/NA1	0.79 (0.48–1.30)	0.354	0.82 (0.43–1.57)	0.545
NA1/NA2	1.00		1.00	
NA2/NA2	1.24 (0.85–1.79)	0.263	1.76 (1.15–2.70)	0.009

^a^Odds Ratio (OR) adjusted with sex and age

^b^Odds Ratio (OR) adjusted with sex, age and Parasite density

### Comparison between the distribution of *FcγRIIIa* genotype and its allelic frequencies among the different study groups

The genotype frequencies showed no statistically significant difference among the *FcγRIIIa*-*F/F* in UM compared to MFC (Table 2). The logistic regression analysis confirmed the absence of significant differences between UM and MFC among the individuals carrying the *FcγRIIIa*-*F/F* genotypes [39% in UM *versus* 45.5% in MFC; OR = 1.95, 95% CI (0.65 to 2.38) and *P* value = 0.79]. Similarly, *FcγRIIIa-V/V* genotype showed no statistically significant association with UM compared to MFC [12.8% in UM *versus* 14.5% in MFC; OR = 1.72, 95% CI (1.04 to 2.82) and *P* value = 0.13] using the heterozygotes as a reference group (Tables 2 and 3). On the other hand, *FcγRIIIa-F/F* genotype was statistically associated with SM compared to UM [72.5% in SM *versus* 39% in UM; OR = 11.51, 95% CI (6.71 to 19.77) and *P* value < 0.001] (Tables 2 and 3). In contrast, the *FcγRIIIa*-*V/V* genotype was statistically negatively associated with SM compared to UM [3.8% in SM *versus* 12.8% in UM; OR = 0.20, 95% CI (0.09 to 0.47) and *P* value < 0.001] (Tables 2 and 3). The frequency analyses also showed differences in the distributions of the heterozygote *FcγRIIIa-F/V* genotype among the three groups (40% in MFC, 48.2% in UM, and 23.6% in SM) (Table 2).

The genotype analyses showed similar frequencies between UM and MFC for carrying the *FcγRIIIb-NA1/NA1* genotypes (Table 2). This was confirmed by logistic regression analysis that revealed the lack of statistically significant difference between these two groups in the *FcγRIIIb-NA1/NA1* [15% in UM *versus* 17.0% in MFC; OR = 0.79, 95% CI (0.48–1.30) and *P* value = 0.354] (Tables 2 and 3). Furthermore, NA2/NA2 genotype was not statistically significantly different among UM patients compared to MFC [36.7% in UM *versus* 37.0% in MFC; OR = 1.24, 95% CI (0.85–1.79) and *P* value = 0.263] using the heterozygotes as a reference group (Tables 2 and 3). Similarly, there was no statistical difference between patients with SM and UM for *FcγRIIIb*-*NA1/NA1* genotype [9.9% in SM versus 15.1% UM; OR = 0.82, 95% CI (0.43 to 1.57) and *P* value = 0.545] (Tables 2 and 3). However, patients carrying the *FcγRIIIb-NA2/NA2* genotype were statistically significantly associated with SM compared to UM [51.6% in SM versus 36.7% in UM; OR = 1.76, 95% CI (1.15- 2.70) and *P* value = 0.009] (Table 2 and 3). The frequency analyses showed differences in the distributions of the heterozygote *FcγRIIIa-NA1/NA2* genotype among the three groups (46% in MFC, 48.2% in UM, and 38.5% in SM) (Table 2).

### Specific IgG subclass reactivity in the different study groups

The antibody response for the *P. falciparum* blood-stage antigen AMA-1 was analysed within the different study groups. The current results showed statistically significant differences among the anti-malarial IgG subclass antibody levels in the different study groups; the overall *P* value < 0.001 (Table 4). In general, the median value of IgG1 and IgG3 subclass were expressed at higher levels than IgG2 and IgG4 antibodies in the UM group when compared to SM subjects (Table 4). To investigate the potential association between the anti-malarial IgG subclass response and protection against infections; the authors first used a logistic regression model to compare the levels of IgG subclasses between the UM infection and MFC (Table 5). The results showed that a higher level of IgG1 against the AMA-1 antigen was associated with UM patients compared to MFC subjects [OR = 1.04; 95% CI (1.01 to 1.07) and *P* value = 0.012]. In addition, the levels of AMA-1-specific IgG3 were significantly higher in UM patients compared MFC [OR = 1.70; 95% CI (1.47 to 1.95) and *P* value < 0.001] (Table 5). There was no observed association for the AMA-1-specific IgG2 and IgG4 responses in UM compared to MFC (Table 5). The second logistic regression model confirmed that the apparent anti-malarial IgG2 and IgG4 antibodies were statistically significantly higher in SM patients when compared to UM patients (Table 5). However, the levels of AMA-1-specific IgG1 and IgG3 were significantly lower in SM group compared to UM patients [for IgG1: OR = 0.89; 95% CI (0.84 to 0.94) and *P* value < 0.001 and for IgG3: OR = 0.52; 95% CI (0.43 to 0.62) and *P* value < 0.001)] (Table 5).

**Table 4:** Comparison of Anti-AMA1 IgG subclasses (µg/mL) among different study groups

Group	IgG1 Median (Q1–Q3)	IgG2 Median (Q1–Q3)	IgG3 Median (Q1–Q3)	IgG4 Median (Q1–Q3)
Malaria-free controls (n = 200)	3.91 (0.2–35.6)	0.89 (0.1–15.1)	1.1 (0.02–15.4)	0.4 (0.1–1.7)
Uncomplicated malaria (n = 218)	4.23 (0.3–47)	0.73 (0.1–43.6)	3.6 (1–29.6)	0.3 (0.1–3.9)
Severe malaria (n182)	2.97 (0.3–25.7)	3.35 (0.3–45.3)	2 (1–8.5)	1.2 (0.2–14.2)
P value*	< 0.001	< 0.001	< 0.001	< 0.001

*AMA-1* Apical membrane antigen 1

^*^P value was derived from *Kruskal–wallis* between the study groups

**Table 5:** *Logistic regression* analysis of malaria specific (anti-AMA1) IgG subclasses levels among the different study groups

Dependent Variable	Model of independent variables		OR (95% CI)	P value
IgG1 AMA1	Uncomplicated malaria	MFC^a^	1.04 (1.01 to1.07)	0.012
		SM^b^	0.89 (0.84 to 0.94)	< 0.001
IgG2 AMA1	Uncomplicated malaria	MFC	1.02 (0.95 to 1.08)	0.640
		SM	1.41 (1.27 to 1.57)	< 0.001
IgG3 AMA1	Uncomplicated malaria	MFC	1.70 (1.47 to 1.95)	< 0.001
		SM	0.52 (0.43 to 0.62)	< 0.001
IgG4 AMA1	Uncomplicated malaria	MFC	1.08 (0.65 to 1.79)	0.77
		SM	15.57 (8.3 to 29.20)	< 0.001

^a^OR represent odds ratios while CI represents confidence intervals. In model (A) uncomplicated versus malaria-free control “MFC”: malaria-free controls were assigned 0 uncomplicated malaria patients were assigned 1 in the logistic regression analysis. OR above 1 represented value higher levels antimalarial IgG subclass associated to uncomplicated malaria while less than 1 value represented malaria-free controls

^b^In model (B) severe malaria versus uncomplicated malaria: uncomplicated malaria was assigned 0 severe malaria patients were assigned 1 in the logistic regression analysis. OR above 1 represented value higher levels antimalarial IgG subclass associated to severe malaria while less than 1 value represented uncomplicated malaria

The results indicated that patients carrying the *FcγRIIa-H/H131* genotype are significantly associated with higher expression levels of the anti-malarial IgG1, IgG2 and IgG4 antibodies, but not IgG3 in UM patients [for IgG1: OR = 0.3; 95% CI (0.2 to 0.6) and *P* value < 0.001, for IgG2: OR = 0.5; 95% CI (0.3 to 0.8) and *P* value = 0.006 and for IgG4: OR = 0.5; 95% CI (0.3 to 0.8) and *P* value = 0.006] (Table 6). Comparatively, patients harbouring the *FcγRIIa*-*R/R131* genotype show significantly increased levels of anti-malarial IgG2 antibodies and associated with SM compared to UM [OR = 3.7; 95% CI (2.0 to 6.7) and *P* value < 0.001] (Table 6). However, patients carrying the genotype *FcγRIIa-R/R131* are statistically negatively associated with higher levels of AMA-1-specific IgG3 [OR = 0.4; 95% CI (0.2 to 0.6) and P value < 0.001] (Table 6). Independently, the model of the multivariate logistic regression analysis of individuals carrying the *FcγRIIIa-F/F* genotype is significantly associated with higher levels of AMA-1-specific IgG2 and IgG4 antibodies in SM compared to UM patients [for IgG2: OR = 3.9; 95% CI (2.4 to 6.4) and *P* value < 0.001 and for IgG4: OR = 3.2; 95% CI (2.1 to 5.3) and *P* value < 0.001] (Table 6). These results together clearly show that the *FcγRIIIa-F/F* genotype is negatively associated with higher expression levels of AMA-1-specific IgG3 among SM compared to UM patients [OR = 0.2; 95% CI (0.1 to 0.4) and *P* value < 0.001] (Table 6). Similarly, our data show that the *FcγRIIIa -V/V* genotype is negatively associated with higher levels of AMA-1-specific IgG4 in SM compared to UM subjects [OR = 0.4; 95% CI (0.2 to 0.6) and *P* value < 0.001] (Table 6).

**Table 6:** *Logistic regression* analysis of individual carrying *FcγRIIa, FcγRIIIa, FcγRIIIb* genotypes in relation of the levels specific IgG subclasses associated with uncomplicated compared to severe malaria

FcγRs genotype s	Antimalrial IgG-AMA-1 subclasses	Genotypes	Adjusted OR^a^ (95% CI)	P value
FcgRIIa	IgG1	R/R131	2.3 (1.4–3.4)	0.485
		H/R131	1	
		H/H131	0.3 (0.2–0.6)	< 0.001
	IgG2	R/R131	3.7 (2.0–6.7)	< 0.001
		H/R131	1	
		H/H131	0.5 (0.3–0.8)	0.006
	IgG3	R/R131	0.4 (0.2–0.6)	< 0.001
		H/R131	1	
		H/H131	1.4 (0.8–2.3)	0.28
	IgG4	R/R131	3.2 (1.8–5.7)	< 0.001
		H/R131	1	
		H/H131	0.5 (0.3–0.8)	0.006
FcγRIIIa	IgG1	F/F	0.8 (0.5–1.2)	0.272
		F/V	1	
		V/V	1.3 (0.8–2.2)	0.326
	IgG2	F/F	3.9 (2.4–6.4)	< 0.001
		F/V	1	
		V/V	0.4 (0.3–0.7)	0.002
	IgG3	F/F	0.2 (0.1–0.4)	< 0.001
		F/V	1	
		V/V	1.8 (0.9–3.7)	0.096
	IgG4	F/F	3.2 (2.1–5.3)	< 0.001
		F/V	1	
		V/V	0.4 (2.0–0.6)	< 0.001
FcγRIIIb	IgG1	NA1/NA1	1.3 (0.7–2.4)	0.47
		NA1/NA2	1	
		NA2/NA2	0.9 (0.6–1.4)	0.71
	IgG2	NA1/NA1	1.0 (0.6–1.9)	0.94
		NA1/NA2	1	
		NA2/NA2	1.4 (0.9–2.1)	0.14
	IgG3	NA1/NA1	0.9 (0.5–1.7)	0.72
		NA1/NA2	1	
		NA2/NA2	0.9 (0.6–1.4)	0.71
	IgG4	NA1/NA1	1.2 (0.6–2.2)	0.61
		NA1/NA2	1	
		NA2/NA2	1.7 (1.1–2.7)	0.011

^a^OR represent odds ratios while CI represents confidence intervals. “OR” adjusted with parasite density. Higher levels of antimalarial IgG subclasses among uncomplicated malaria patients were assigned 0 while higher levels antimalarial IgG subclasses among severe malaria patients were assigned 1 in the logistic regression analysis. OR above 1 represented value associated to higher levels antimalarial IgG subclasses among severe malaria patients while less than 1 value represented higher levels antimalarial IgG subclasses among uncomplicated malaria patients

Furthermore, the analyses of the results show that the *FcγRIIIb-NA2/NA2* genotype is significantly associated with a higher level of AMA-1-specific IgG4 among SM compared to UM group [OR = 1.7; 95% CI (1.1 to 2.7) and *P* value = 0.011] (Table 6). The current results indicate that the *FcγRIIIb* genotypes are not associated with the independent action of the three IgG subclasses (IgG1, IgG2, and IgG3) of antibodies, and maybe due to the absence of interaction in the logistic regression model.

## Discussion

This study aimed to evaluate the possible relationship between the variants of *FcγRIIa* (CD32a), *FcγRIIIa* (CD16a), *FcγRIIIb* (CD16b) gene polymorphism and *P. falciparum* AMA-1-specific IgG subclass and its importance in the susceptibility to complicated malaria infections among children in Saudi Arabia. This study is the country's first report investigating this association among children.

The data of this investigation suggested that there was no significant impact of the *FcγRIIa-R/H131* genotypes polymorphism on the susceptibility to UM infection compared to MFC. This finding is in parallel with the previously published report from Eastern Sudan by Giha and co-workers, which suggested the lack of statistically significant association between *FcγRIIa*-*R/H131* genotypes polymorphism on immunity and susceptibility to UM infection [25]. This may be due to the similarities in malaria epidemiology, malaria transmission, and patient’s semi-immunity to malaria infection [3, 16]. In contrast, the study of Shi et al*.* demonstrated a protective effect against UM for *FcγRIIa-R/R131* compared with the heterozygote *FcγRIIa*-*R/H131* genotype carriers in infants below one year of age [26]. Therefore, there is no general agreement regarding the role of *FcγRIIa*-*R/H131* in UM infection. In the present study, the logistic regression model suggests that the genotypes *FcγRIIa*-*R/R131* are statistically significantly associated with increased susceptibility to SM infection (2.1-fold) when compared to UM patients. In contrast, the current data indicates that the *FcγRIIa-H/H131* is negatively associated with SM (0.349-fold decrease) compared to UM patients. Similarly, in a former study, the authors have reported that the *FcγRIIa-R/R131* genotypes are associated with SM, while the *FcγRIIa*-*H/H131* genotypes show a significant association with mild malaria among Sudanese patients residing in East Sudan [9]. Previously published case–control investigations have demonstrated that *FcγRIIa-R/R131* genotypes are associated with protection against high parasite density [26], and the genotypes of *FcγRIIa*-*H/H131* are correlated with high risk of either severe malaria or placental malaria [27–29]. Interestingly, the current study found that the levels of IgG1, IgG2, and IgG4 are associated with *FcγRIIa*-*H/H131* in the UM patients. Nasr et al*.* have suggested similar results among the Fulani ethnic group that are less susceptible to severe malaria infection [11]. In contrast, previous data on pregnant women with asymptomatic malarial infection (ASM) revealed that the high levels of AMA-1-specific anti-malarial IgG1, IgG2, and IgG4 antibodies are statistically associated with R/R131 carriers rather than the genotype *FcγRIIa*-*H/H131* [3]. This contradiction may be due to the variation in the individual’s genetic background, and variation in study designs.

The results of this study suggest that the relative reduction in malaria infection in the UM group cannot be explained solely by the magnitude and quality of the humoral response to malaria. Additional studies are needed to clarify whether the *FcγRIIa*-*R/H131* polymorphism is a causative factor in the variable predisposition to malaria that is demonstrated among the different groups.

This study also revealed that the *FcγRIIIa*-*F/V176* genotypes are not associated with UM patients compared to MFC. On the other hand, the *FcγRIIIa*-*F/F176* genotype is statistically associated with SM compared to UM patients. However, patients carrying the *FcγRIIIa-V/V176* genotypes are statistically associated with protection against SM compared to UM. The latter finding is in line with a recent Kenyan study, which shows that the polymorphisms in the *FcγRIIIa-V/V* are associated with protection against severe malaria and modulations in circulating IFNγ levels [12]. In contrast, a previous investigation on Thai patients did not show an association between *FcγRIIIa-F/V176* genotypes and the severity of the disease [29]. Again, these discrepancies may be attributed to the difference in ethnicity and study design.

The current study suggests that individuals carrying the *FcγRIIIa*-*F/F* genotype are significantly expressing higher levels of AMA-1-specific IgG2 and IgG4 antibodies in the SM group compared to patients with UM. In agreement with this finding, Koene et al*.* have shown that the *FcγRIIIa*-*F/F* is significantly less bound to IgG1, IgG3, and IgG4 compared to the *FcγRIIIa*-*V/V* genotypes [30].

The study’s results suggest that there are no statistically significant differences between UM and MFC for the *FcγRIIIb*-*NA1/NA1* and *FcγRIIIb*-*NA2/NA2* genotypes. In contrast, the patients carrying the *FcγRIIIb-NA2/NA2* genotype are significantly associated with SM compared to patients with UM. Recent work on children living in Western Kenya suggests that the *FcγRIIIb-NA1/NA2* gene polymorphisms are not significantly associated with susceptibility to severe malaria [12]. In addition, the study performed by Adu et al*.* have demonstrates that the *FcγRIIIb-NA2/NA2* in Ghanaian children is associated with clinical malaria [4]. In 2010, Adu demonstrated an association between the *FcγRIIIb*-*NA2/NA2* and susceptibility to severe and uncomplicated malaria among Ghanaian children [31]. These contradicting results may be attributed to the different malaria transmission seasons and malaria epidemics. Moreover, different ethnicity associated with variations in the genetic background may significantly contribute to the *FcγR* gene polymorphism and susceptibility/protection to severe malaria [11].

### Strength and limitations

To the best of the authors knowledge, this is the first study in the Kingdom of Saudi Arabia which highlighted the relation between *FcγR* genotypes polymorphism, IgG subclass and malaria infection among Saudi children. This will hopefully lead to further research in the area. Some of the studies limitations include the small sample size and the fact that the study was performed in one region of Saudi Arabia instead of it being multi-centred. As such findings need to be confirmed in a large sample size from various regions representing the whole endemic area.

## Conclusion

This study reveals significant influence of the *FcγRIIa-R/R131, FcγRIIIa-F/F176* and the *FcγRIIIb-NA2/NA2* genotypes in increasing the susceptibility to severe malaria. Binding between the FcγR genotypes and IgG subclass results in changes in the ability of the immune cells to respond to infection through secretion of inflammatory mediators during *P. falciparum* infection. Further studies are underway in our laboratory to elucidate if the *FcγRIIa, FcγRIIIa* and *FcγRIIIb* genotypes polymorphism contribute to the differential susceptibility to malaria among the different study groups.

## Data Availability

The datasets used and/or analysed during the current study are available from the corresponding author upon request.

## Abbreviations

AMA-1: Apical membrane antigen 1
AMPSJ: Aledabi Malaria Prevention Station in Jazan
ASRED: Allele specific restriction enzyme digestion
BMGHJ: Bani Malik General Hospital in Jazan
FcyR: Fc-gamma receptors
PCR: Polymerase chain reaction
